# Informing wind energy development: Land cover and topography predict occupancy for Arizona bats

**DOI:** 10.1371/journal.pone.0268573

**Published:** 2022-06-03

**Authors:** Clarissa A. Starbuck, Brett G. Dickson, Carol L. Chambers

**Affiliations:** 1 School of Forestry, Northern Arizona University, Flagstaff, Arizona, United States of America; 2 Lab of Landscape Ecology and Conservation Biology, Landscape Conservation Initiative, Northern Arizona University, Flagstaff, Arizona, United States of America; Southeastern Louisiana University, UNITED STATES

## Abstract

Wind energy is a growing source of renewable energy with a 3-fold increase in use globally over the last decade. However, wind turbines cause bat mortality, especially for migratory species. The southwest United States has high bat species diversity and is an important area for migratory species, although little is known about their seasonal distribution. To examine potential risk to bats in areas proposed for wind energy development, we characterized bat occupancy spatially and temporally across northern Arizona, identifying use during summer when bats are reproductively active and fall during the migratory season. Our objectives were to determine occupancy of migratory species and species of greatest conservation need and develop a probability of occupancy map for species to identify areas of potential conflict with wind energy development. We selected 92 sites in 10 clusters with potential for development and used acoustic detectors to sample bats in the summer and fall of 2016 and 2017 for 6 nights per site per year. We predicted response of migratory bat species and species of special concern to 9 landscape variables using Program MARK. During summer, higher densities of forest on the landscape resulted in a higher probability of occupancy of migratory species such as hoary bats (*Lasiurus cinereus*), silver-haired bats (*Lasionycteris noctivagans*), big free-tailed bats (*Nyctinomops macrotis*), and species of conservation need such as spotted bats (*Euderma maculatum*). During the fall, higher concentration of valleys on the landscape predicted occupancy of hoary bats, big free-tailed bats, and spotted bats. High bat occupancy in the fall was also associated with higher elevation and close proximity to forests. We recommend that wind turbines be placed in open, flat grasslands away from forested landscapes and concentrations of valleys or other topographic variation.

## Introduction

The use of wind energy has increased worldwide >3 fold in the past decade [1], and the capacity in the United States (~100 Gigawatts [GW]) accounted for almost a fifth of global wind energy capacity in 2018 [2]. Although wind turbines are a clean, renewable source of energy, collisions with turbines can be fatal to many bat species [3–7]. Indeed, as humans increase their use of renewable sources of energy, bat fatalities caused by wind turbines have become a threat to bat populations, globally. From 1790 to 2000, only 3% of multiple mortality events (≥10 dead bats in a locality in <1 year; MMEs) for bats were attributed to fatalities at wind turbines; however, after 2000, these fatalities increased to nearly 35% and were the leading cause of MMEs for bats [8]. The number of wind turbines in the United States increased over 12-fold after 2000. There were 4,675 wind turbines built from 1980 to 1999 and 58,888 turbines were built from 2000–2021 [9].

Globally, the species that are most affected by wind turbines appear to be those that fly and forage in open environments [10, 11]. Both migratory and resident bats are killed at wind turbines, and some studies suggest that wind energy facilities can kill bats from local and distant populations [12, 13]. Globally, 41 species were reported killed at wind energy facilities [8]; however, this probably underestimates species affected. In areas of greatest bat diversity, such as the tropics, there are likely to be many more species affected by wind turbines, but there are few studies of effects of wind energy on bats from these areas [11].

In North America, migratory species (hoary bats [*Lasiurus cinereus*], silver-haired bats [*Lasionycteris noctivagans*], eastern red bats [*Lasiurus borealis*], and Mexican free-tailed bats [*Tadarida brasiliensis*]) are killed at higher rates at wind turbines than non-migratory species [14, 15] due to their preference for open-air flight, or possible attraction to wind turbines [6, 16]. Long-distance flying species such as the spotted bat (*Euderma maculatum*), a species of conservation concern in Arizona [17], might also be affected. The nightly flight patterns of spotted bats include many vegetation types, such as open grassland and scrubland where wind turbines have been built [18]. Frick et al. [19] determined that even with optimistic estimates, there could be as much as a 90% population decline for hoary bats in the next 50 years because of fatalities at wind turbines. Piorkowski and O’Connell [20] found that Mexican free-tailed bats constituted most fatalities at a wind energy facility in Oklahoma, and this species accounts for most fatalities at wind turbines in the southwest (33.1%) and Pacific southwest (52.6%) regions of the United States [21]. Without mitigation to reduce fatalities at existing wind energy facilities [22] and understanding of how bats use areas prior to construction of wind facilities, mortalities will increase annually as more wind turbines are constructed [14].

Understanding how bats use landscape features during migration can inform the placement of turbines to decrease mortality. Other landscape-scale occupancy maps using acoustics have shown that bat occupancy is predicted by landcover and landform features [23, 24], but these maps have not been made for migratory bats in the southwest United States. In other parts of North America and Europe, migrating bats fly in concentrated areas along specific topographic positions [25, 26] and appear to use large topographic landforms, such as mountain ranges, to help guide them during long distance movements [27, 28]. Other studies focused on smaller scales in the western United States have predicted distance to cliffs and canyons [18, 29, 30], elevation [31–33], aspect and slope [31], vegetation type [18, 32], and water density or distance to water [31–34] are important to bats, and we know that migratory bats move across the United States-Mexico border between summer and winter [35]. Given the topographically rich region and potential corridor for bat movement in the southwestern United States, this is an important area during migration for migratory species affected by wind turbines: hoary bats, silver-haired bats, and Mexican free-tailed bats [28, 35]. However, only one study in the southwestern United States [36] has examined how geophysical characteristics of turbine placement affects bat mortality at a wind energy site. They found that turbines placed closer to escarpment edges had higher bat mortalities [36], but this study was at one wind energy site where all turbines were placed relatively close to an escarpment edge.

Our objectives were to determine seasonal use of landscape features by migratory bats and species of conservation concern in Arizona and develop a map predicting occupancy along a potential flight path for bats in the southwest United States. We focused on migratory species that were most affected by wind turbines in other parts of the United States (hoary bat and silver-haired bat), fly high above the ground in open areas (Mexican free-tailed bat and big free-tailed bat), or were species of conservation concern (spotted bat) that could be differentiated using echolocation calls. We hypothesized that bats would use large topographic landforms (e.g., cliffs, escarpments) and vegetation (e.g., forest cover) to guide large-scale movements. Specifically, we predicted that bats would have higher occupancy closer to: 1. forests because they provide roosting opportunities for hoary bats and silver-haired bats, and forest edges also provide more foraging opportunities than open grasslands and scrublands [37, 38], 2. water because bats use water sources for foraging or drinking [e.g., 39], and water is a limiting resource in the arid southwestern United States, and 3. topographic features, such as valleys and mountains, because they may aid navigation during migration for these species or are foraging and roosting opportunities. Our goal was to find areas with specific landscape characteristics in migratory regions that had low bat occupancy and high wind classes that would provide opportunities for wind energy development while avoiding collisions and mortalities of bats at wind turbines.

## Materials and methods

### Study area

**Fig 1:**
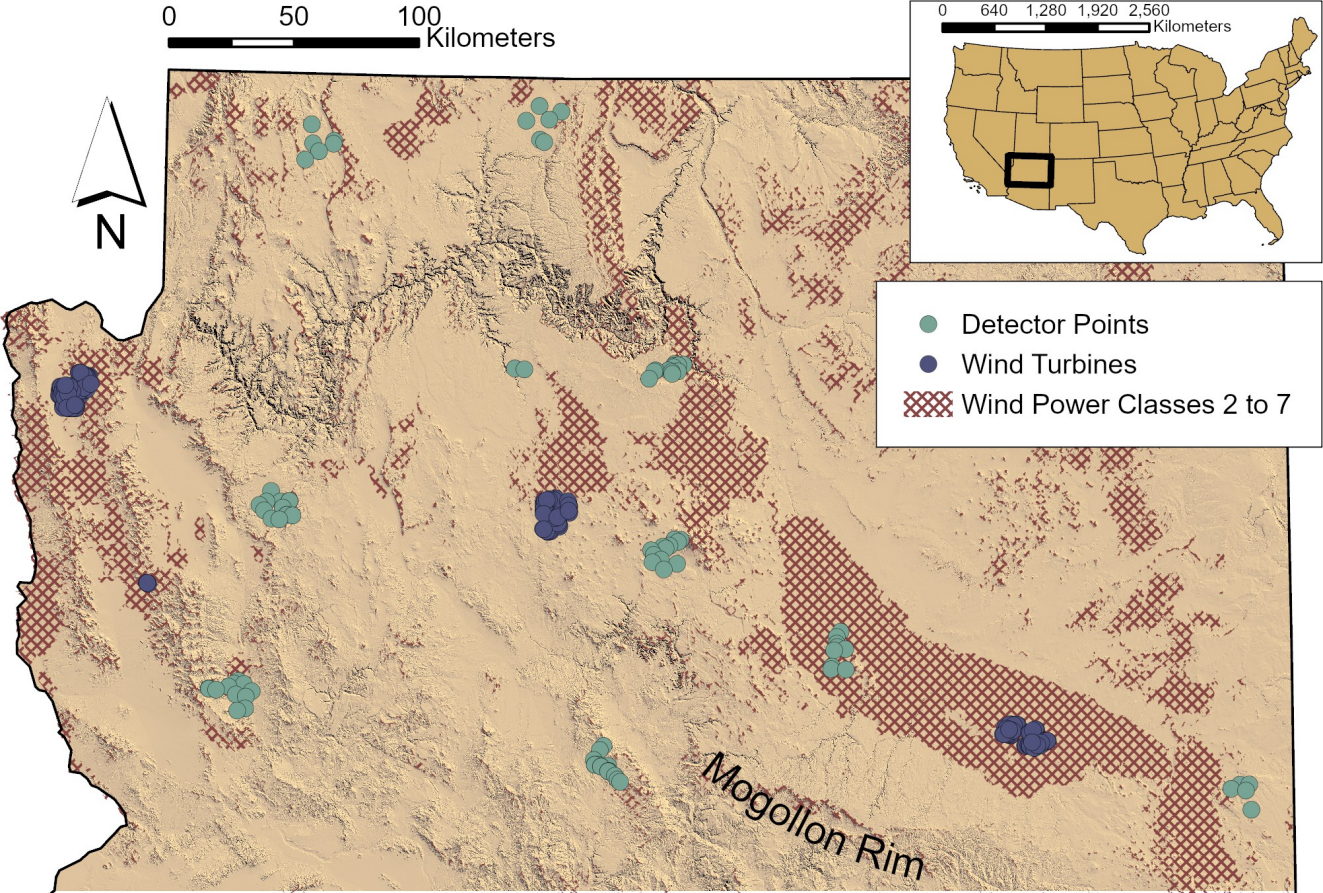
Study area showing detector locations, wind turbine locations, and wind power classes 2 to 7 in northern Arizona. Wind power classes 2 to 7 in northern Arizona are represented by the hatched area. Wind power classes range from 1 to 7 and represent a mean wind speed at a certain height above the ground, with 1 the lowest wind speed and 7 the highest wind speed [41]. Acoustic detector locations are green circles, and existing wind turbines are blue circles [9]. The Mogollon Rim represents the southern boundary of the study area.

We selected northern Arizona as our 16,202,300-hectare study area. It was bounded by Arizona state borders on the north, east, and west, and the Mogollon Rim to the south (Fig 1). Northern Arizona consists of four main biotic communities: Petran montane conifer forest (ponderosa pine [*Pinus ponderosa*] and a mix of other conifers such as Douglas-fir [*Pseudotsuga menziesii*], spruce [*Picea* spp.], and fir [*Abies* spp.]), Great Basin conifer woodland (pinyon pine [*Pinus edulis*] and juniper [*Juniperus* spp.]), plains and Great Basin grassland (mixed grasses and shrubs), and Great Basin desertscrub (mixed shrubs; [40]). We surveyed bats in areas that were characteristically similar to current wind energy developments in northern Arizona. These areas include open grasslands, scrublands, or shrublands. Wind power classes range from 1 to 7 and represent a mean wind speed at a certain height above the ground, with 1 the lowest wind speed and 7 the highest wind speed [41]. Although sites with a wind power class rating of ≥4 are most often selected for development, in Arizona current wind developments occur on sites with a wind power class ≥1 (G. Ritter, Arizona Game and Fish Department, personal communication). We attempted to select sites in wind power classes to match current wind energy developments, however because of land access we had more sites in lower wind classes than what was representative of the wind turbines. Many areas with higher wind power classes were on private land with difficult or no access. We used the National Land Cover Database [42] to determine and select land cover classes. Elevation ranged from 655 to 2636 m. Our study period extended from 15 June to 24 November 2016 and 16 June to 16 November 2017 to capture the summer maternity period and the fall migratory period when most fatalities from wind turbines have occurred in North America. The mean temperature and precipitation in Flagstaff, Arizona (NAD83, 12 N 440713E, 3895202N) during our study period was 18.9 ± 0.82°C and 0.25 ± 0.07 cm in the summer (15 June to 15 August) of 2016, 0.35 ± 0.77°C and 0.13 ± 0.05 cm in the fall (15 September to 24 November) of 2016, 19.49 ± 0.80°C and 0.28 ± 0.09 cm in the summer (16 June to 15 August) of 2017, and 9.59 ± 0.86°C and 0.00 ± 0.00 cm in the fall (15 September to 16 November) of 2017 [43].

### Acoustic recording

We deployed SongMeter 3 Bat (SM3Bat) echolocation detectors (Wildlife Acoustics, Inc., Maynard, MA) across 10 distinct site clusters; each cluster had radius of 10 km. We chose to use site clusters for two reasons: 1. there is high variability of bat movement, even within short distances [44], and 2. the feasibility of conducting the study with limited personnel and funding. We used a geographic information system (GIS; ArcGIS 10.2.2 ESRI, Redlands, CA, USA) to randomly select each cluster in grassland and scrubland cover types on land managed by the U. S. Forest Service, Bureau of Land Management, or private landowners. Within each cluster, we randomly located 10 sites ≥500 m apart. Not all sites were surveyed each year, and detectors failed at some sites, so not all sites were used in analysis. Sites were ≥1 km but <5 km from water to avoid biasing detections and ≤200 m from a road for ease of access. At each site, we set up a detector so that the microphone was attached to the top of an 8-m aluminum pole secured with three supporting ropes. At this height, we could detect bats flying within the lower rotor sweep zone of a wind turbine built to current specifications in northern Arizona. The SM3Bat microphones detect low frequency (≤30 kHz) bats from 23 to 39 m (Detecting Bats with Ultrasonic Microphones, https://www.wildlifeacoustics.com/uploads/publications/UltrasonicMicrophones.pdf, Wildlife Acoustics, 2014), and wind turbine blades in northern Arizona reach approximately 35 to 38 m from the ground [9]. We used omnidirectional SM3-U1 microphones and tested microphone sensitivity with a Wildlife Acoustics ultrasonic microphone calibrator before placement to make sure that the sensitivity was consistent across each detector unit. The microphone was angled 45^o^ downwards to prevent moisture affecting the microphone. We used four D-cell batteries to power each of the detectors. Detectors recorded at each site from 30 minutes before sunset to 30 minutes after sunrise each night for ≥6 consecutive nights per season (summer: 15 June to 15 August, fall: 15 September to 15 November). A site was sampled in either 2016 or 2017, but not both years. Summer dates were chosen to reflect the reproductive period [45], and fall dates represented the period during which bats are migrating [46]. We separated each period by a month so that they were distinct. The detectors recorded sounds >12 dB for 5 seconds; each echolocation call file was 5 seconds long. We classified a call sequence as a 5-sec file with ≥2 echolocation pulses. Echolocation call files were stored on removable SD memory cards while in the SM3Bat detector and then downloaded to a computer for analysis.

We used SonoBat scrubber to remove non-bat calls and SonoBat (SonoBat version 3.2.1, Arcata, CA) to identify the calls to species. In the SonoBat Batch Classify tool, we used a decision threshold of 0.95, acceptable call quality of 0.80, acceptable quality to tally passes of 0.20, and maximum number of calls to consider per file of 8. After the calls were classified in SonoBat, we manually vetted the calls to species using SonoBat and AnalookW version 4.2n (C. Corben, www.hoarybat.com). All calls were transformed to zero-cross files using Kaleidoscope version 4.3.2 (Wildlife Acoustics, Inc., Maynard, MA) for viewing in AnalookW. In AnalookW, we manually compared calls to known reference calls and call characteristics of species in the southwestern United States (R. Mixan, Arizona Game and Fish Department, personal communication).

### Landscape variables and analyses

We examined relationships between bat occupancy and 8 landscape variables that we hypothesized to be related to occupancy (Table 1). We determined which spatial scale was most meaningful to our species of interest by including variables at multiple spatial scales [47–49]. We calculated values at each site for each variable using a GIS, where each data layer had a pixel size of 30 x 30 m. For each landscape variable, we used a moving window analysis in the GIS to determine values for variables that required calculations of an area around each site at 7 spatial scales (90, 180, 360, 720, 1440, 2880, and 5760 m radius). These scales were systematically selected because they represented local (e.g., 90 to 720 m) to landscape (≥1440 m) habitat use by bats (e.g., [33, 49, 50]). This range in spatial scales helped us to identify patterns at the appropriate scale for each species. Other researchers have found that bats respond to predictors at multiple scales, ranging from 90 m to 32 km [33, 49, 51]. We used the National Land Cover Database (NLCD) [42] to determine forested areas. We used this database to measure distance from a site to the nearest forested pixel and the percent of forested pixels around a site at each of 7 spatial scales. We used a landform data layer that consisted of 15 landform classes to determine the landform type [52]. The valley landform type represented riparian areas and canyons that provide connectivity for bats on the landscape, and the cliff landform type represented steep rock faces that could provide roosting and foraging areas for bats. We separated the valley and cliff landform type from the rest of the dataset to calculate percent of pixels classified as valley around a site within each of 7 spatial scales on the landscape and to calculate distance (m) from a site to cliffs. We calculated stream density (m/m^2^) and distance to lakes (m) using stream and lake datasets from the Arizona State Land Department (https://land.az.gov/maps-gis-0). We used a digital elevation model to determine elevation and slope at each site. Range in elevation was calculated from a digital elevation model as the range in meters in elevation around each site at the 7 spatial scales. Some variables were underrepresented (i.e., contained >50% zero values at local spatial scales), so those variables were not used at those scales. We also calculated topographic position index at 360 m around each site using the equation from Theobald et al. [52]. Topographic position index (TPI) is the measure of relative topographic relief where peaks and ridges are highly positive values, flat lands are represented by values near zero, and valley bottoms are represented by highly negative values [52]. We used this scale because it performed the best of all spatial scales in preliminary univariate analyses. All values of each variable were standardized to z-scores ($\bar{x}$ = 0, SD = 1) before being included in models.

**Table 1:** Landscape variables used in the models to predict bat occupancy in northern Arizona. Landscape variables for the study sites, their minimum, maximum, mean, and standard deviation (SD) used to predict bat occupancy in a study in northern Arizona, USA, from June to November of 2016 and 2017.

Variable		Minimum	Maximum	Mean	SD
Percent Forest					
	90 m	0.00	100.00	5.93	17.21
	180 m	0.00	100.00	8.82	20.69
	360 m	0.00	100.00	11.68	23.59
	720 m	0.00	96.99	14.23	26.55
	1440 m	0.00	98.43	16.37	28.87
	2880 m	0.00	91.23	18.45	29.80
	5760 m	0.00	89.18	22.51	30.04
Distance to forest (m)		0.00	11621.90	1696.10	2377.39
Elevation (m)		655.00	2636.00	1634.94	445.76
Stream density (m/m^2^)					
	2880 m	0.00	0.74	0.11	0.15
	5760 m	0.00	0.42	0.15	0.09
Distance to lake (m)		1087.06	25295.10	10213.43	5768.31
Distance to cliffs (m)		1188.70	14060.40	6001.94	2751.27
Topographic Position Index		-1.04	1.95	0.08	0.55
Percent Valley					
	360 m	0.00	42.34	5.79	9.45
	720 m	0.00	23.39	5.15	6.24
	1440 m	0.00	20.76	5.25	4.64
	2880 m	0.44	15.34	5.99	3.22
	5760 m	0.84	11.86	6.59	2.58
Range in elevation (m)					
	90 m	2.00	50.00	11.45	9.88
	180 m	2.00	87.00	21.22	18.15
	360 m	6.00	197.00	40.36	33.19
	720 m	12.00	260.00	72.71	50.13
	1440 m	24.00	409.00	138.60	90.10
	2880 m	51.00	879.00	260.92	158.91
	5760 m	118.00	1581.00	509.22	294.07

We used single-season occupancy modeling [53] in program MARK [54] to predict each bat species response to landscape variables. We held occupancy (Ψ) constant to determine the best model for predicting probability of detection (*p*), and then used those variables for *p* to evaluate the best models of Ψ. We used Akaike’s Information Criterion adjusted for small sample sizes (AICc) to rank models in MARK and to select the model that best explained the data [55]. We used Julian date, maximum daily temperature (°C), minimum daily temperature (°C), and daily precipitation (cm) from the Western Regional Climate Center [43] as detection covariates (*p*). We considered distance to forest (m), elevation (m), range in elevation (m), distance to lakes (m), distance to cliffs (m), slope (degrees), percent forest, stream density (m/m^2^), percent valley, year, and cluster as variables for occupancy. We tested variables for collinearity and did not use variables in the same model if they were correlated. We first performed a univariate analysis to determine the best fitting spatial scale for each variable for each species (e.g., [49]). Only the best fitting scale (i.e., lowest AICc value) was used in further analysis for that species, and we only included variables in multivariate models for that species if the univariate model had an AICc less than the null model [55]. We used the most parsimonious model to assess model fit using the MacKenzie-Bailey goodness-of-fit test [56] in program PRESENCE (version 2.13.6) [57]. We included models with ΔAICc ≤4 in the candidate set of models, and we calculated the model averaged coefficients, unconditional standard errors, and relative variable importance (*w*_*ij*_) for each covariate [55, 58]. Covariates with a relative variable importance value ≥0.50 suggested a strong species response, <0.50 and ≥0.30 had a moderate species response, and <0.30 had a weak species response (e.g., [59]).

We created predictive maps for our study area using the GIS. For each species, we created the map using the model-averaged estimates of variables that were in the candidate model set for each season.

## Results

**Fig 2:**
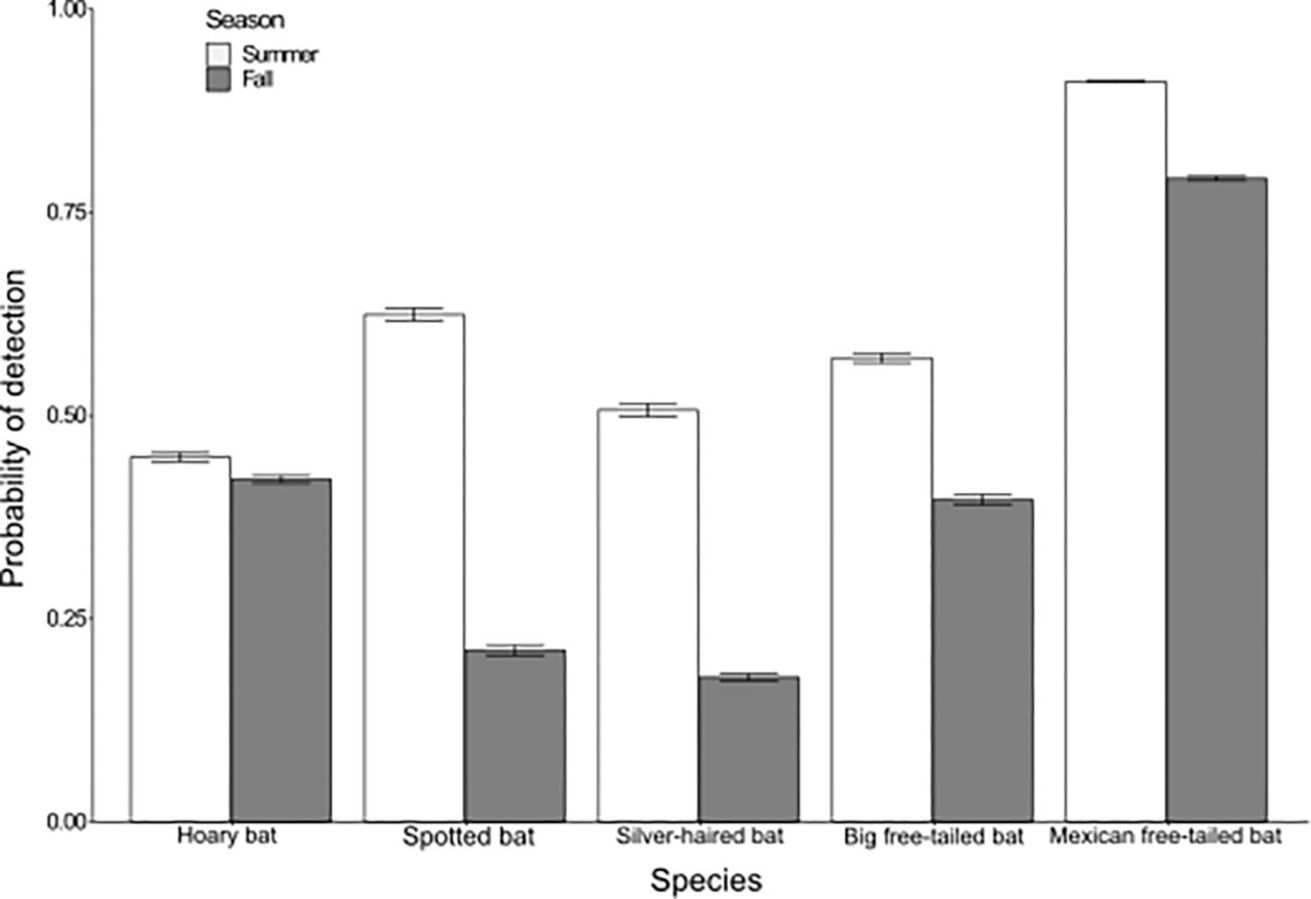
Probability of detection for 5 bat species in northern Arizona. Probability of detection during summer and fall of 5 bat species (hoary bat [*Lasiurus cinereus*], spotted bat [*Euderma maculatum*]), silver-haired bat [*Lasionycteris noctivagans*], big free-tailed bat [*Nyctinomops macrotis*], and Mexican free-tailed bat [*Tadarida brasiliensis*]) with standard error bars in northern Arizona, USA, from June to November in 2016 and 2017.

We recorded bat activity at 56 sites in 2016 and 36 sites in 2017, using 6 nights per site to create detection histories. Compared to wind turbines currently in northern Arizona, we had a higher percentage of sites in wind power class 1 and a lower percentage of sites in higher wind power classes—wind power class 1: our sites = 75.0%, turbines = 15.4%; wind power class 2: our sites = 20.7%, turbines = 68.1%; wind power class 3: our sites = 4.3%, turbines = 15.4%; wind power class 4: our sites = 0.0%, turbines = 1.2%. We collected 304,346 bat calls over summer and fall of 2016 and 2017, detecting all species of interest (Mexican free-tailed bats, hoary bats, silver-haired bats, big free-tailed bats, and spotted bats). Average probability of detection was higher in the summer than the fall for all species (Fig 2). Neither year nor cluster affected bat occupancy, so they were not included in the final analyses. The probability of occupancy for 4 of 5 species had a positive relationship with percent forest on the landscape or distance to forest.

Mexican free-tailed bats were detected at 100% of sites in summer and fall, so the parsimonious model showed poor fit for predicting Mexican free-tailed bat occupancy (summer ĉ = 5.25, P-value = 0.02; fall ĉ = 6.59, P-value = 0.00). The best model for estimating *p* was maximum daily temperature in the summer and daily precipitation and Julian day in the fall, however the null model was included in the candidate set (Table 2). The null model for occupancy was the best model for predicting summer and fall occupancy for Mexican free-tailed bat, thus no landscape variable predicted occupancy.

**Table 2:** Models of bat occupancy in summer and fall across northern Arizona. The log likelihood (LogLike), Akaike Information Criteria difference values (ΔAICc), and weight (*w*_*i*_) for each of the models with difference values ≤4.0 and the null model [p(.)psi(.)] for each species (hoary bat [*Lasiurus cinereus*], silver-haired bat [*Lasionycteris noctivagans*], big free-tailed bat [*Nyctinomops macrotus*], and spotted bat [*Euderma maculatum*]) in each season (summer and fall) for an occupancy study conducted from 15 June to 24 November 2016 and 16 June to 16 November 2017 in northern Arizona, USA.

Model^a^			LogLike	ΔAICc	*w*_*i*_
Mexican free-tailed bat					
	Summer				
		p(Tmax)psi(.)	279.92	0.00	0.15
		p(Precip)psi(.)	280.17	0.25	0.13
		p(Date)psi(.)	281.20	1.28	0.08
		p(.)psi(.)	283.55	1.47	0.07
		p(Tmax)psi(Forest5760)	279.92	2.22	0.05
		p(Tmax)psi(ForestDist)	279.92	2.22	0.05
		p(Tmax)psi(Elevation)	279.92	2.22	0.05
		p(Tmax)psi(Stream5760)	279.92	2.22	0.05
		p(Tmax)psi(LakeDist)	279.92	2.22	0.05
		p(Tmax)psi(CliffDist)	279.92	2.22	0.05
		p(Tmax)psi(Slope)	279.92	2.22	0.05
		p(Tmax)psi(Valley5760)	279.92	2.22	0.05
		p(Tmax)psi(Elev5760)	279.92	2.22	0.05
		p(Tmax)psi(Year)	279.92	2.22	0.05
		p(Tmax)psi(TPI360)	279.92	2.22	0.05
		p(Tmin)psi(.)	283.43	3.51	0.03
	Fall				
		p(Precip,Date)psi(.)	399.87	0.00	0.18
		p(Date)psi(.)	402.92	0.78	0.12
		p(Precip,Date)psi(Elev2880)	399.87	2.34	0.06
		p(Precip,Date)psi(Forest5760)	399.87	2.34	0.06
		p(Precip,Date)psi(ForestDist)	399.87	2.34	0.06
		p(Precip,Date)psi(Elevation)	399.87	2.34	0.06
		p(Precip,Date)psi(Stream5760)	399.87	2.34	0.06
		p(Precip,Date)psi(LakeDist)	399.87	2.34	0.06
		p(Precip,Date)psi(CliffDist)	399.87	2.34	0.06
		p(Precip,Date)psi(Slope)	399.87	2.34	0.06
		p(Precip,Date)psi(Valley5760)	399.87	2.34	0.06
		p(Precip,Date)psi(Year)	399.87	2.34	0.06
		p(Precip,Date)psi(TPI360)	399.87	2.34	0.06
		p(.)psi(.)	408.03	3.70	0.03
Hoary bat					
	Summer				
		p(Tmax)psi(Forest5760,TPI360)	536.83	0.00	0.90
		p(.)psi(.)	580.42	36.93	0.00
	Fall				
		p(Precip)psi(Forest360,Valley720)	462.11	0.00	0.74
		p(.)psi(.)	480.12	11.22	0.00
Silver-haired bat					
	Summer				
		p(Tmax)psi(Forest5760,Slope)	416.65	0.00	0.47
		p(Tmax)psi(Forest5760,Elevation)	418.32	1.66	0.21
		p(Tmax)psi(Forest5760)	420.92	1.98	0.18
		p(Tmax)psi(Elevation,Elev5760,Slope)	417.91	3.60	0.08
		p(.)psi(.)	482.46	59.14	0.00
	Fall				
		p(Tmax)psi(Elevation,ForestDist)	248.42	0.00	0.67
		p(Tmax)psi(Elevation,Stream5760)	250.27	1.85	0.26
		p(.)psi(.)	288.42	33.21	0.00
Big free-tailed bat					
	Summer				
		p(Tmax)psi(Forest5760,Valley5760)	389.34	0.00	0.58
		p(Tmax)psi(Forest5760)	392.30	0.68	0.41
		p(.)psi(.)	443.82	47.82	0.00
	Fall				
		p(Date)psi(ForestDist,Elev5760)	446.25	0.00	0.91
		p(.)psi(.)	472.38	19.34	0.00
Spotted bat					
	Summer				
		p(Tmin)psi(Elevation,Valley2880)	236.79	0.00	0.72
		p(Tmin)psi(Forest5760,Valley2880)	239.05	2.25	0.23
		p(.)psi(.)	274.75	31.29	0.00
	Fall				
		p(Date)psi(Valley5760,ForestDist)	260.00	0.00	0.52
		p(Date)psi(Valley5760)	262.99	0.65	0.37
		p(Date)psi(Elevation,ForestDist,CliffDist)	261.31	3.72	0.08
		p(.)psi(.)	289.16	22.36	0.00

^a^ Variables for the models included: Precip = daily precipitation, Tmin = minimum daily temperature (°C), Tmax = maximum daily temperature (°C), Date = Julian day, Year = the year the data were collected, Slope = slope (in degrees), TPI360 = Topographic Position Index at 360 m surrounding the site, LakeDist = distance to nearest pond or lake (m), ForestDist = distance to nearest forest (m), CliffDist = distance to nearest cliff (m), Elevation = elevation (m), Valley = percent of pixels surrounding a site that were classified as valley, Forest = percent of forest surrounding a site, Stream = stream density around a site (m per m^2^), Elev = range in elevation around a site. The number following the variable name is the scale (in meters) that was selected as the most appropriate scale for that variable for that species in the univariate models.

We detected hoary bats at 76% of sites in summer and 75% of sites in fall. There was no evidence for lack of fit for summer or fall models (summer ĉ = 1.15, P-value = 0.19; fall ĉ = 1.25, P-value = 0.09). The best model for estimating *p* in the summer included maximum daily temperature; the best model in the fall included minimum and maximum daily temperature (Table 2). Percent forest at 5760 m and TPI both had a strong, positive effect on hoary bat occupancy (Table 3), although confidence intervals crossed zero for percent forest. There was one candidate model for estimating fall Ψ (Table 2). Hoary bat occupancy increased strongly with percent valley at 720 m and percent forest at 360 m (Table 3), but confidence intervals crossed zero for percent forest.

**Table 3:** Variables included in each candidate model for 4 bat species in northern Arizona. The model averaged estimate, unconditional standard error (*SE*), 95% confidence intervals (CI), and relative variable importance (*w*_*ij*_) for each variable included in the candidate models for species in an occupancy study of 4 bat species (hoary bat [*Lasiurus cinereus*], silver-haired bat [*Lasionycteris noctivagans*], big free-tailed bat [*Nyctinomops macrotus*], and spotted bat [*Euderma maculatum*]) in northern Arizona, USA, from June to November in 2016 and 2017.

Model		Variable and scale	Estimate	*SE*	Lower CI	Upper CI	*w*_*ij*_
Hoary bat							
	Summer						
		Forest at 5760 m	1.83	1.04	-0.21	3.88	0.96
		TPI	2.01	1.02	0.02	4.02	0.92
		Intercept	1.81	0.71			
	Fall						
		Valley at 720 m	1.95	8.60	0.27	3.63	0.90
		Forest at 360 m	1.45	0.81	-0.12	3.04	0.76
		Intercept	2.31	0.67			
Silver-haired bat							
	Summer						
		Forest at 5760 m	1.35	0.63	0.11	2.59	0.85
		Slope	-1.10	1.21	-3.46	1.28	0.55
		Elevation	0.28	0.44	-0.58	1.14	0.35
		Range in elevation at 5760 m	0.51	1.00	-1.44	2.47	0.11
		Intercept	0.06	0.58			
	Fall						
		Elevation	7.15	5.15	-2.89	17.24	0.99
		Distance to Forest	-4.36	4.09	-12.33	3.66	0.67
		Stream density 5760 m	-0.40	0.64	-1.64	0.85	0.27
		Intercept	0.80	1.00			
Big free-tailed bat							
	Summer						
		Forest at 5760 m	1.88	0.57	0.77	3.00	0.98
		Valley at 5760 m	-0.44	0.50	-1.42	0.55	0.59
		Intercept	0.36	0.36			
	Fall						
		Distance to forest	-3.40	1.30	-5.93	-0.86	0.94
		Range in elevation at 5760 m	-10.20	3.74	-17.49	-2.88	0.93
		Intercept	1.09	0.53			
Spotted bat							
	Summer						
		Valley at 2880 m	1.18	0.41	0.38	1.99	0.95
		Elevation	1.19	0.75	-0.26	2.65	0.75
		Forest at 5760 m	0.25	0.40	-0.52	1.03	0.24
		Intercept	-1.30	0.36			
	Fall						
		Valley at 5760 m	2.15	1.06	0.08	4.23	0.89
		Distance to forest	-1.11	1.33	-3.72	1.50	0.61
		Distance to cliff	-0.38	0.75	-1.84	1.09	0.10
		Elevation	-0.13	0.24	-0.60	0.35	0.09
		Intercept	0.54	0.79			

**Fig 3:**
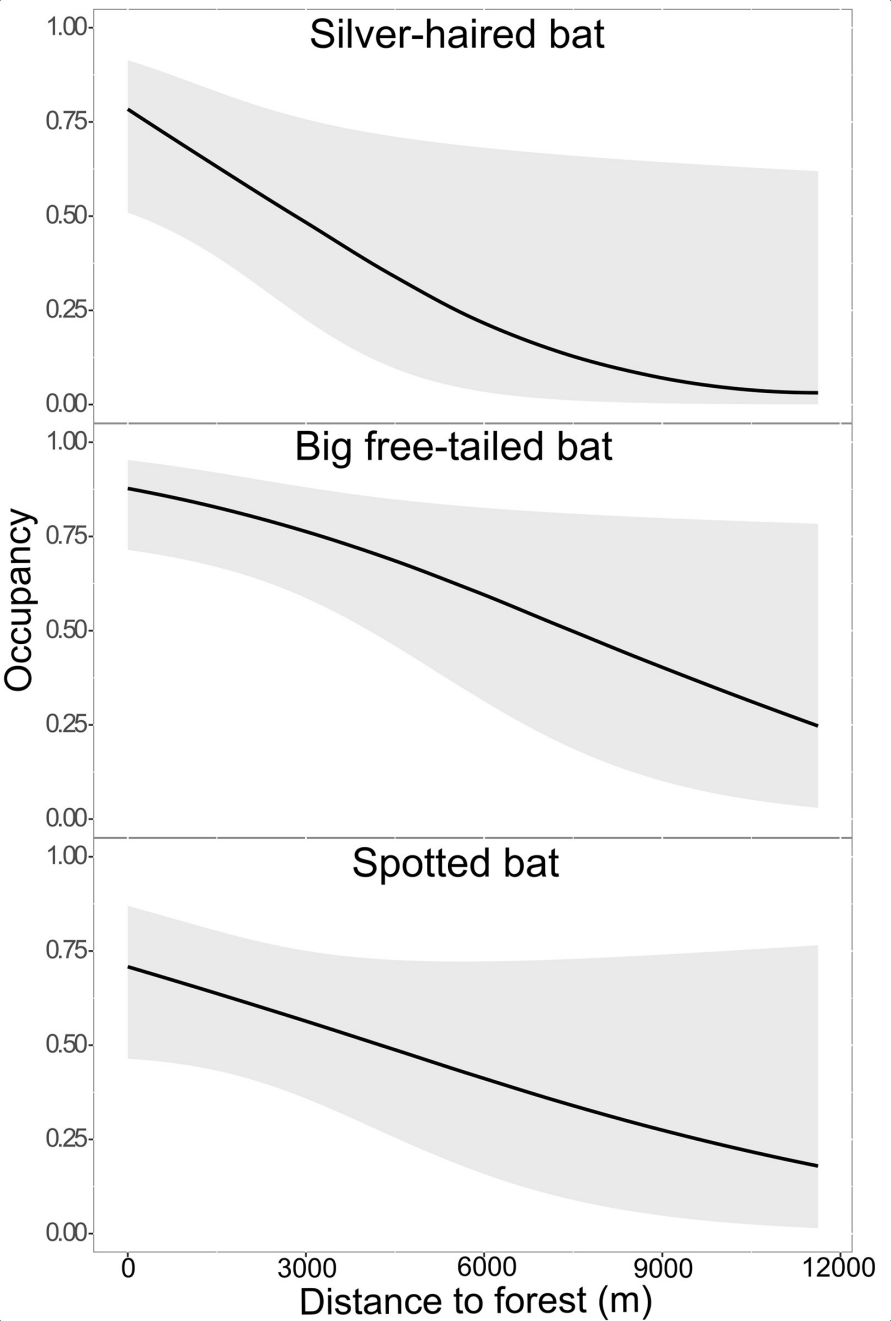
The probability of occupancy relative to distance to forest (m) for 3 species in northern Arizona. Probability of occupancy relative to distance to forest (m) during fall for silver-haired bat (*Lasionycteris noctivagans*), big free-tailed bat (*Nyctinomops macrotis*), and spotted bat (*Euderma maculatum*) in northern Arizona, USA, from June to November in 2016 and 2017. We recommend that wind turbines are placed ≥6 km away from forests to avoid areas of high (>0.60) bat occupancy.

We detected silver-haired bats at 58% percent of points in the summer and 42% of points in the fall. The models showed no evidence of lack of fit (summer ĉ = 0.93, P-value = 0.41; fall ĉ = 0.87, P-value = 0.61). In both summer and fall, detection of silver-haired bats increased with maximum daily temperature (Table 2). Silver-haired bat occupancy had a positive relationship with elevation, percent forest at 5760 m, and range in elevation at 5760 m, but a negative relationship with slope in the summer (Table 3). Relative variable importance was strong for percent forest at 5760 m and slope, moderate for elevation, and weak for range in elevation at 5760 m (Table 3). Confidence intervals crossed zero for slope, elevation, and range in elevation. In the fall, silver-haired bat occupancy increased strongly with an increase in elevation and decreased strongly with distance to forest (Fig 3, Table 3) but decreased weakly with higher stream density at 5760 m (Table 3). However, confidence intervals crossed zero for all variables.

Big free-tailed bats were detected at 53% of the points in the summer and 72% of the points in the fall. There was no evidence for lack of fit (summer ĉ = 1.35, P-value = 0.10; fall ĉ = 1.15, P-value = 0.20). Big free-tailed bat detection decreased as maximum daily temperature increased in the summer (Table 2). Big free-tailed bat occupancy had a strong positive relationship with percent forest at 5760 m and a strong negative relationship with percent valley at 5760 m (Table 3), but confidence intervals crossed zero for percent valley. In the fall, probability of detection for big free-tailed bats was higher at the beginning than at the end of the season (Table 2). Big free-tailed bat occupancy in the fall decreased strongly with distance to forest (Fig 3, Table 3) and range in elevation at 5760 m (Table 3). Occupancy of big free-tailed bats increased with more forest and fewer valleys on the landscape in the summer, and in the fall big free-tailed bat occupancy increased closer to forests and with more elevation range.

Spotted bats were detected at 29% of the points in the summer and 42% of points in the fall. The summer models showed no evidence for lack of fit (ĉ = 0.49, P-value = 0.98), however the fall models showed some evidence for lack of fit (ĉ = 1.78, P-value = 0.03). In the summer, spotted bat detection increased as minimum daily temperature increased (Table 2). Spotted bat occupancy increased strongly in the summer with elevation, percent valley at 2880 m, and increased weakly with percent forest at 5760 m (Table 3), and confidence intervals crossed zero for elevation and percent forest. In the fall spotted bat detection decreased with an increase in Julian day (i.e., fewer spotted bats detected in November than in September; Table 2). Spotted bat occupancy in the fall increased with percent valley on the landscape at 5760 m, decreased farther from forests, farther from cliffs, and at higher elevations (Table 3). Relative variable importance for percent valley on the landscape at 5760 m and distance to forest was strong (Fig 3, Table 3); however, relative importance for distance to cliff and elevation was weak (Table 3). Confidence intervals for distance to forest, distance to cliff, and elevation crossed zero. In the summer, spotted bat occupancy was greater at higher elevations, and with more valleys and forest on the landscape. In the fall, spotted bat occupancy was higher with more valleys on the landscape and closer to forests and cliffs at higher elevations.

**Fig 4:**
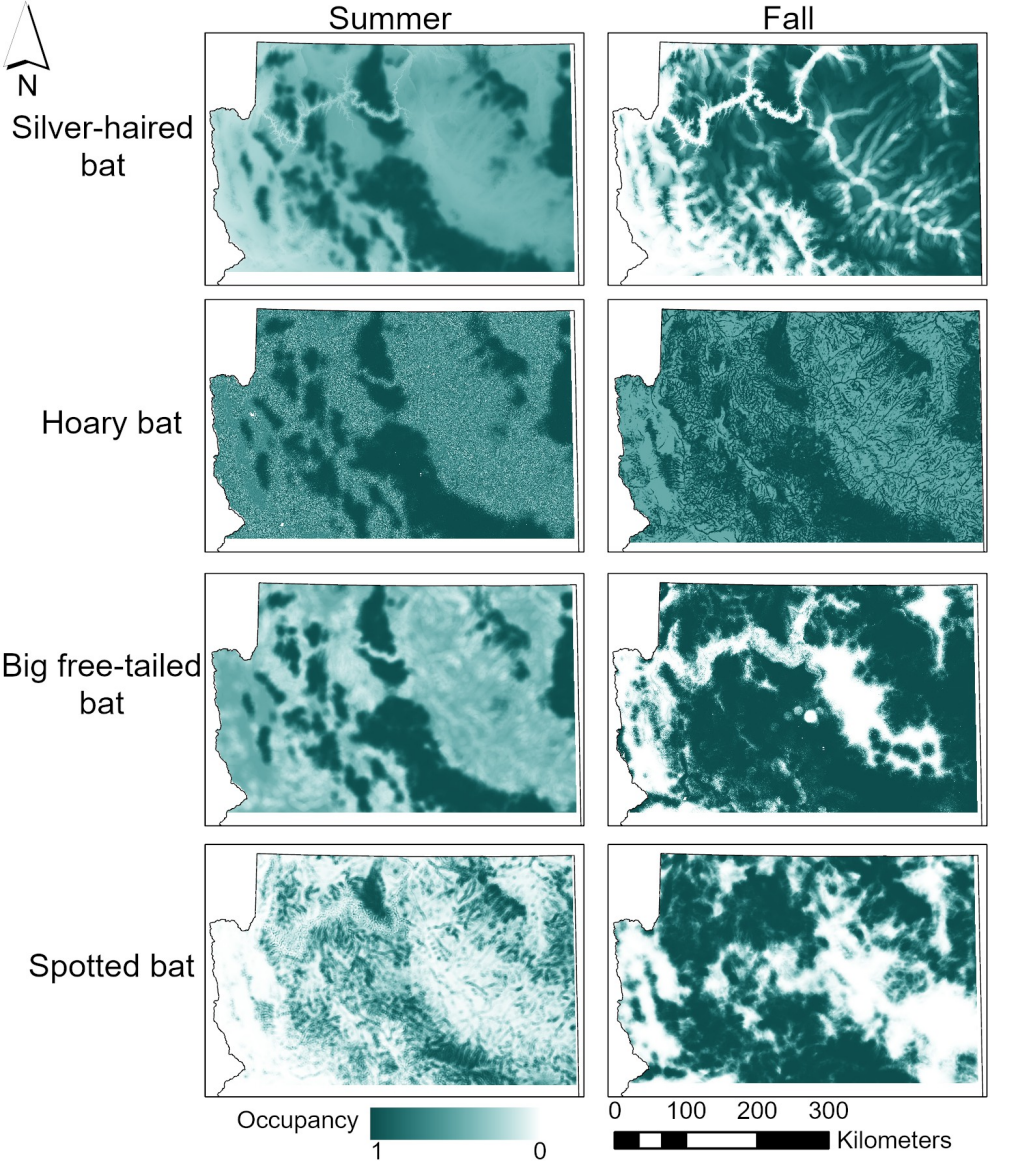
The probability of occupancy during summer and fall across northern Arizona for 4 bat species. Probability of occupancy during summer and fall of 4 bat species (silver-haired bat [*Lasionycteris noctivagans*], hoary bat [*Lasiurus cinereus*], big free-tailed bat [*Nyctinomops macrotis*], and spotted bat [*Euderma maculatum*]) in northern Arizona, USA, from June to November in 2016 and 2017.

**Fig 5:**
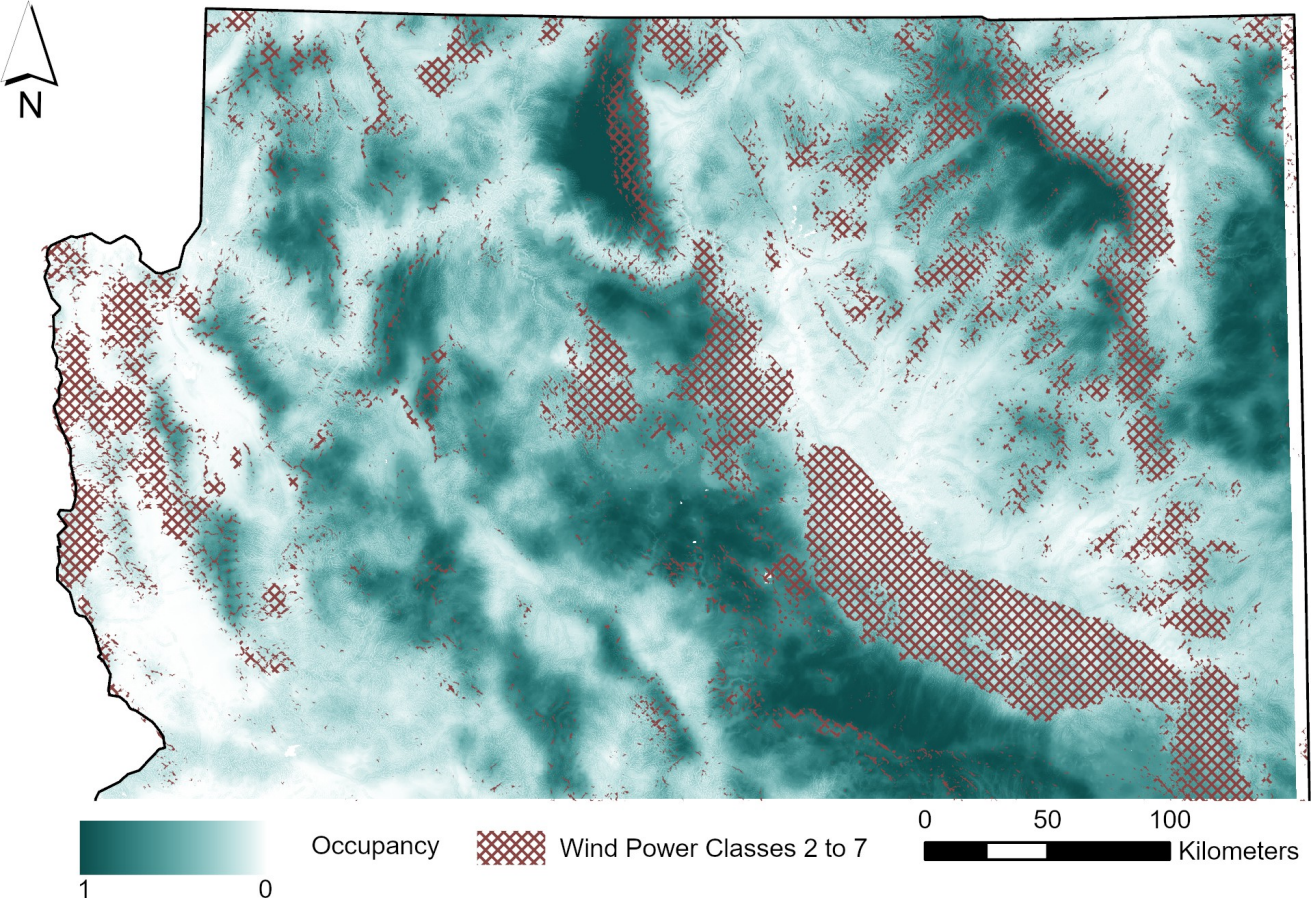
Probability of co-occurrence across northern Arizona for 4 bat species. The probability that 4 bat species (silver-haired bat [*Lasionycteris noctivagans*], hoary bat [*Lasiurus cinereus*], big free-tailed bat [*Nyctinomops macrotis*], spotted bat [*Euderma maculatum*]) co-occur in northern Arizona, USA, from June to November in 2016 and 2017. Wind power classes 2 to 7 in northern Arizona are indicated by hatching, where wind power classes range from 1 to 7 and represent a mean wind speed at a certain height above the ground, with 1 the lowest wind speed and 7 the highest wind speed [41].

Maps depicting probability of occupancy in the summer for the 4 species with models showed high probability of occupancy primarily along the Mogollon Rim, a 320-km topographic and geological feature, which covers the south-central part of the study area and forms the southern edge of the Colorado Plateau in Arizona (Figs 4 and 5). In the fall, these 4 species generally had higher occupancy across the study area, so bats were more broadly dispersed across northern Arizona than in the summer (Fig 5).

## Discussion

Most species in our study had higher occupancy near forests, water, and topographic features, supporting our predictions. Migratory bats and those that fly high above ground in open areas in the southwest United States could be affected by wind energy development, especially if turbines are placed near forested, topographically variable landscapes with valleys and water sources.

We found some support that forests are important for migratory bats and species of special concern in both the summer and fall. Forests are used by many bat species in summer as roosting and foraging areas. Hoary bats and silver-haired bats roost in large diameter trees [60–63]. Hoary, silver-haired, and spotted bats also forage along forest edges [18, 37, 38] and use ponderosa pine forests more than more open land cover types, such as pinyon pine (*Pinus* spp.) and juniper (*Juniperus* spp.) woodlands in western New Mexico [64]. In their review of bat mortality at wind energy facilities in the United States and Canada, Thompson et al. [65] found that higher bat mortality was associated with less grassland cover and more forest cover surrounding the turbine. In Portugal, Santos et al. [66] also found that higher bat mortality at wind energy facilities occurred <5 km from forests. Other species forage in or above forests, for example, in northern Arizona, the big free-tailed bat and spotted bat fly from roosting sites in cliffs to forested foraging areas in the summer [18, 29, 50]. Forests provide roosts and foraging areas for multiple bat species, and wind turbines should be placed away from forests to reduce bat fatalities.

We found that higher elevations may be important for silver-haired bats in the fall and spotted bats in the summer. Cooler temperatures at higher elevations may provide thermal relief for bats as they forage during the summer. For example, female spotted bats roost at warm, lower elevations but leave their pups to fly long distances to higher elevations to forage at cooler temperatures [18, 50]. Foraging at sites with cooler temperatures also allows individuals to conserve energy by using short torpor bouts to drop their body temperatures while night roosting [50, 67]. Bats can then have more capacity for flight, an energetically expensive activity [68]. Non-reproductive individuals (e.g., males and non-reproductive females) are able to tolerate cooler temperatures and are generally captured more often at higher elevations than reproductive females [39, 67, 69]. Male silver-haired bats may use high elevation sites more during the fall than female silver-haired bats, and in northern Arizona, 95% of silver-haired bats captured are males (C. Chambers, Northern Arizona University, unpublished data).

Our results suggested that valleys were more important to hoary bats in the fall than in the summer. Linear landforms such as mountain ranges, escarpments, and valleys may be used as navigational landmarks for bats during migration. Baerwald and Barclay [25] found higher activity of hoary bats close to mountain ranges than in the open plains in Alberta, Canada, and Rydell et al. [10] found higher bat mortality at wind turbines that were closer to topographic features such as ridges, than on flat open farmland. These landforms can also provide foraging opportunities for bats. We found that valleys were important for spotted bats in both the summer and fall. Spotted bats use canyons as flyways to higher elevation foraging areas in northern Arizona [18, 50], but spotted bats and big free-tailed bats also forage along cliff edges [18, 29]. Spotted bats could also be using valleys as foraging areas in northern Arizona.

Although we saw some differences between summer and fall, our recommendations for siting wind turbines are the same for both seasons. Forest was an important predictor for both seasons for some species, and distance to forest was especially important in the fall for big free-tailed bats. Although confidence intervals were large, there was a clear decline in bat occupancy as distance to forest increases (Fig 3). This effect was greatest for big free-tailed bats in the fall, so we use this species to make recommendations and suggest that wind turbines be placed at least 6 km from forests to avoid areas with high (≥60%) probability of bats occurring.

New wind turbines are constructed with taller towers and longer blades [10, 70, 71]. The average for turbine hub height and rotor diameter in 2018 was 88.1 m and 115.6 m, respectively, but turbines built in 2019 reached total heights of >180 m [9]. As the height of turbines increases, we might expect a greater negative effect on high-flying species such as Mexican free-tailed bat. Our microphones were deployed 8 m above ground, so it is likely that we missed individuals that flew higher. However, we achieved detection probabilities >0.4 for all 5 bat species in the summer and for 3 of 5 bat species in the fall. An occupancy study at a wind energy facility in southern California had similar detection probabilities (<0.4) for all microphone heights (2 m, 22 m, and 52 m above ground) except for one period [72], so we believe that our results are comparable to those that had microphones at higher heights. Other studies that described bat mortality at wind energy facilities found similar relationships to our acoustic occupancy study (i.e., higher mortality closer to forests and topographic landforms; [10, 66]), so our outcome and recommendations would probably not differ using microphones at higher heights. Other researchers have suggested that resource availability or pre-construction monitoring does not predict bat mortality at the wind energy site [73–75]. Bats could be attracted to wind turbines for foraging, mating, or out of curiosity [6, 16, 76, 77]. However, Bennett and Hale [74] looked at only one site in their study, and most pre-construction monitoring measures bat activity (i.e., number of bat calls/time), which is highly variable and does not predict bat fatality rates [75]. We also conducted our study across a landscape scale and looking at bat occupancy instead of activity across a larger area may better predict areas that are more suited for wind energy facilities, although there are currently no pre-construction monitoring studies that measured bat occupancy.

Effects to some bat species could be reduced by siting turbines away from areas of high bat occupancy, but ubiquitous species, such as the Mexican free-tailed bat, will be affected throughout the southwest. For ubiquitous species and for bats that may be attracted to wind turbines [6, 16, 76, 77], other mitigation measures can reduce bat fatalities after wind turbine construction such as increasing the wind speed at which turbines turn on (cut-in speed) [22].

Understanding how bats use the landscape at multiple sites and at scales that reach ≥5 km from proposed wind energy sites is an important first step to identifying proper siting of wind energy development facilities to reduce bat mortality at these facilities. Mitigation at currently operating turbines such as increasing the cut-in speed will minimize bat fatalities of some bat species [22] and does not have a large effect on energy production [78]. Looking at landscape scales, such as in our study, and determining relationships with bat occupancy on that landscape will help improve placement of wind energy facilities. Focusing on important migratory pathways, such as the southwest United States, will also help protect bats as they move through to other areas of their range.
